# Supplementary material for: Molecular Typing of Listeria monocytogenes IVb Serogroup Isolated from Food and Food Production Environments in Poland
Source: Pathogens. 2021 Apr 15;10(4):482. doi: 10.3390/pathogens10040482 (PMC8071568; doi:10.3390/pathogens10040482)
Supplement: Supplementary file 1 [file pathogens-10-00482-s001.zip › Supplementary Materials/FIGURE S1.pdf]

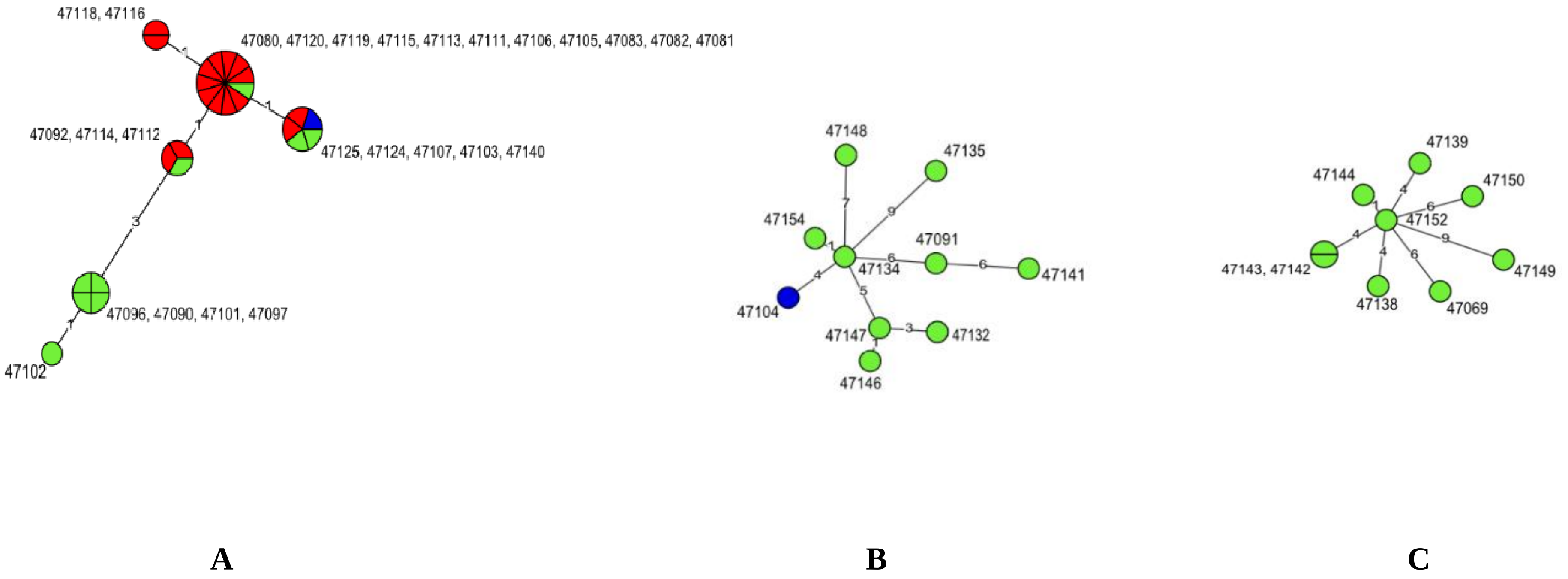

FIGURE S1. Minimum spanning tree (MST) analysis based on cgMLST profiles (CTs) of 45 *L. monocytogenes* belonging to three CTs: A, CT375; B, CT4325; C, CT4380. The different colors indicate isolate source (green, ready-to-eat food; red, raw meat; blue, food production environments). Numbers on the connecting lines show alleles differences between adjacent nodes (CTs). The numbers next to circles show ID of the currently tested *L. monocytogenes* strains.
